# PDB_Amyloid: an extended live amyloid structure list from the PDB

**DOI:** 10.1002/2211-5463.12524

**Published:** 2018-11-22

**Authors:** Kristóf Takács, Bálint Varga, Vince Grolmusz

**Affiliations:** ^1^ PIT Bioinformatics Group Eötvös University Budapest Hungary; ^2^ Uratim Ltd. Budapest Hungary

**Keywords:** amyloid, amyloid‐precursor, human chorionic gonadotropin, PDB, thymidylate synthase, web server

## Abstract

The Protein Data Bank (PDB) contains more than 135 000 entries at present. From these, relatively few amyloid structures can be identified, since amyloids are insoluble in water. Therefore, most amyloid structures deposited in the PDB are in the form of solid state NMR data. Based on the geometric analysis of these deposited structures, we have prepared an automatically updated web server, which generates a list of the deposited amyloid structures, and also entries of globular proteins that have amyloid‐like substructures of given size and characteristics. We have found that by applying only appropriately selected geometric conditions, it is possible to identify deposited amyloid structures and a number of globular proteins with amyloid‐like substructures. We have analyzed these globular proteins and have found proof in the literature that many of them form amyloids more easily than many other globular proteins. Our results relate to the method of Stanković *et al*. [Stanković I *et al*. (2017) IPSI BgD Tran Int Res 13, 47–51], who applied a hybrid textual‐search and geometric approach for finding amyloids in the PDB. If one intends to identify a subset of the PDB for certain applications, the identification algorithm needs to be re‐run periodically, since in 2017 on average 30 new entries per day were deposited in the data bank. Our web server is updated regularly and automatically, and the identified amyloid and partial amyloid structures can be viewed or their list can be downloaded from the following website https://pitgroup.org/amyloid.

**Abbreviations**
hCG: human chorionic gonadotropin
PDB: Protein Data Bank
TS: thymidylate synthase A

The Protein Data Bank (PDB) is a continually developing public resource of spatial structures of proteins and nucleic acids 1. Today the database contains more than 135 000 structures. The geometric properties of these molecules can be analyzed by bioinformatical tools, and one may infer significant new relations in these very complex macromolecular structures through such analyses 2, 3, 4, 5, 6, 7, 8, 9. Here, we are interested in amyloid structures in the Protein Data Bank.

Amyloids are misfolded protein aggregates that are present in numerous biological structures including the cellular surface of a number of microorganisms 10, 11, where they have a role in host–pathogen interaction; the silkmoth chorion and some fish choria, forming protective films 12; the immune system of certain insects, helping in the encapsulation of pathogens and parasites 13; healthy human pituitary secretory granules, for storing peptide hormones 14; and human amyloidoses and several neurodegenerative diseases 15. Amyloid structures sometimes show prion‐like infective properties 15, 16, 17. Cerebral β‐amyloid plaques have long been considered to be biomarkers of Alzheimer's disease 18, 19, 20, 21, although more recently their validity has been challenged by several authors 22, 23, 24.Mechanisms of amyloid formation are reviewed in 25, 26. Amyloid fibers are formed from parallel β‐sheets, with hydrogen bonds between the parallel strands. It is widely accepted that amyloid formation requires the presence of a nucleus or a seed of amyloid‐forming segments with exposed edges of β‐sheet structures 25, 26, 27, 28.

Since amyloid fibers are insoluble in water, until very recently there were no high‐resolution structures deposited in the RCSB PDB 1. Today, one can find several dozen atomic‐resolution amyloid structures in the PDB, and this dataset has opened up the possibility of the analysis and the data mining of the properties of these misfolded proteins, using their high‐resolution spatial structure.

The first step in this direction is the identification of the amyloid structures in the PDB.

Amyloid and amyloid‐precursor molecules have been collected and predicted using protein‐sequencing data in numerous articles (e.g. in the AMYPdb resource 29, or in 30). We are interested in the analysis of the spatial protein structures for finding amyloid and amyloid‐precursor molecules, rather than in the analysis of residue‐sequence properties of proteins of unknown three‐dimensional structure.

In a remarkable piece of work, Stanković *et al*. 31 screened the PDB for amyloid structures by applying the following procedure: (a) by a textual search, those PDB entries were selected that contain the word ‘amyloid’ or any of another 38 words describing amyloid precursors; (b) helical structures, identified by torsion angles, were discarded; and (c) parallel, near‐linear fragments of length at least four residues were identified; structures without these fragments were also discarded.

In the present work, we prepared an automatically updated list of amyloid and potentially amyloidogenic structures from the PDB, applying only the geometric properties of β‐sheets; consequently, we did not use any textual search, referring to the annotations of the PDB entries. By this choice, we intended to identify not only the aggregated amyloid entries and known precursors, but also those globular proteins that contain small, locally amyloid‐like substructures. We assumed that these globular proteins may also be amyloidogenic, i.e. they can more easily turn into amyloid fibers than globular proteins without these structural elements.

Since the PDB grows very quickly – in 2017, every day on average 30 new structures were deposited – we needed to construct an automatically updated web server, which periodically examines the new PDB entries and includes the newly deposited amyloid and potentially amyloidogenic structures. Consequently, our list does not give a snapshot of the amyloid structures in the PDB at a given time as with other efforts, but rather presents a live list of these structures. Our online resource is available at https://pitgroup.org/amyloid/.

## Materials and methods

Here we describe the selection method that generates the Extended Amyloid List at https://pitgroup.org/amyloid/.

In contrast with Stanković *et al*. 31, we did not make any selection through a textual search in the annotation fields of the PDB files. Instead, we attempted to collect the minimal set of geometric rules, which already return the amyloids found in 31, plus novel, globular proteins with possible amyloidogenic substructures.

We constructed three rules, (a)–(c), in which, informally, rule (a) assures the parallelism of the β‐sheets; rule (b) excludes the structures with large curvature, e.g. locally parallel helices; and rule (c) ensures that the approximately straight and parallel fragments are sufficiently long, compared with the total length of the chain. More formally, the following rules were applied:

- For finding parallel β‐sheets. Stanković *et al*. 31 selected parallel chain segments by requiring the distance difference between the closest *C*_α_ atoms of the fragment to be less than 1.5 Å. Instead of this condition, we have applied a limit to the standard deviation σ between the closest *C*_α_ atoms of the fragment such that its value is less than 1.5 Å. We think that this approach is more tolerant of singular, random errors in the structure, while it is strict enough to characterize the parallel polypeptide chains in the amyloid structures. More technically, our condition can be re‐phrased as follows. Let us consider two separate chains of the structures, *A* and *B*, both identified as β‐sheets. Next, we compute the array *C*(*A*)_dist_, which contains the minimum distance for every *C*_α_ atom of chain *A* from the closest *C*_α_ that is located in chain *B*. Next, we identify the maximal subchains *F* of *A*, satisfying σ(*C*(*F*)_dist_) ≤ 1.5, while every distance in vector *C*(*F*)_dist_ is required to be between 2 and 15 Å.
- Excluding structures with large curvature. Stanković *et al*. 31 excluded helical structures from consideration. We apply a locally verifiable angular condition for the fragments *F* as follows. Fragment *F*, which satisfies the conditions in (a), needs also to satisfy the condition that the angles of each of three consecutive *C*_α_ atoms, averaged for the fragment *F*, need to be between 110° and 180°. In other words, these angles, on average, should be obtuse angles between 110° and 180°.
- Condition for the minimum length of parallel fragments. len(*F*) ≥ len(A)/7, where *F* denotes the same as in rule (a), and len(*X*) denotes the length of chain *X*, measured in residues.

The specific parameters for the conditions above were selected for including all multi‐chain amyloid structures that were also found in Stanković *et al*. 31. We did not aim to find amyloid‐like structures containing only one single polypeptide chain since the amyloid structures contain a large number of approximately parallel fibers, each consisting of different chains. While the PDB contains partial amyloid structures with one single chain (e.g. 1HZ3), these structures will not be listed within our results, since they lack the characteristic property of nearly parallel, distinct polypeptide chains. The search for distinct parallel chains is useful for disallowing single chains with long parallel subsections, for example, β‐barrel structures, such as the bacterial porin structure 4RLC. Since amyloid aggregates always consist of a large number of distinct, approximately parallel polypeptide chains, our condition is not restrictive.

## Results and discussion

### Amyloid structures

We have found that our list at https://pitgroup.org/amyloid/ contains all amyloid structures with at least two polypeptide chains that are listed by Stanković *et al*. 31. For example, the classical amyloid structures of 2KIB, 2N0A, 5KO0, 2LBU and 2LMN are all present in the list.

### Possible amyloidogenic structures

Here, we review four non‐amyloid proteins that were found by our screening algorithm and that are listed at https://pitgroup.org/amyloid/. We also give literature evidence showing links to the amyloid formation of these molecules. These findings are witness to the power of our algorithm, but clearly we cannot review here the more than 500 structures presented on the webpage https://pitgroup.org/amyloid/.

**Figure 1:**
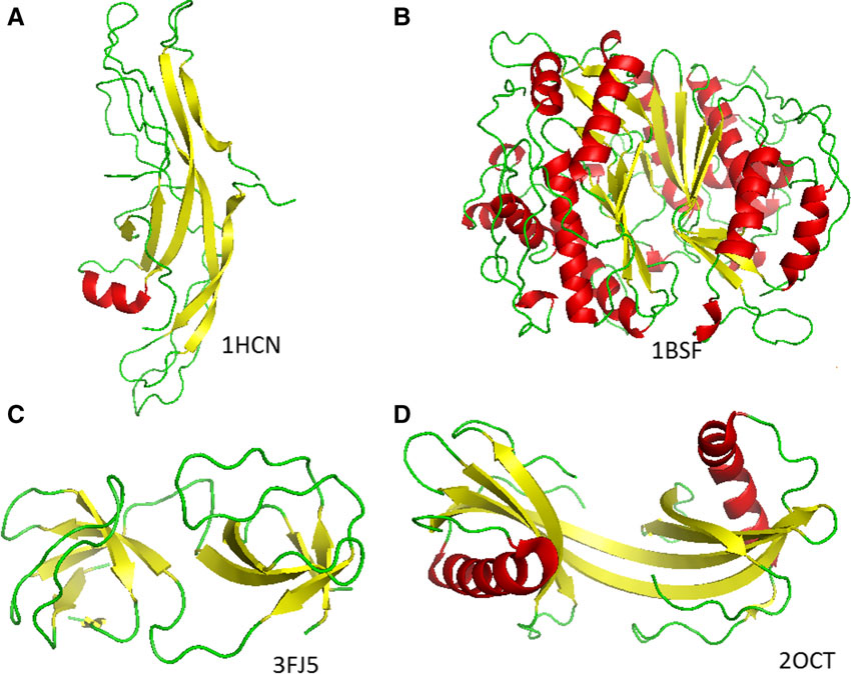
Protein structures 1HCN, 1BSF, 3FJ5 and 2OCT, with partial amyloid‐like substructures. All of these entries are documented as amyloidogenic in the literature.
1BSF: Thymidylate synthase A (TS) from *Bacillus subtilis* (Fig. 1B). Thymidylate synthase has an important role in DNA synthesis, and its aggregational properties have been studied for a long time 36. The human TS is a primary target of cancer chemotherapy, most importantly by 5‐fluorouracil, a strong‐binding TS inhibitor, applied widely in colon, esophageal, stomach, pancreatic, breast and cervical cancers. In Fig. 1B, it can be clearly seen that the parallel β‐sheets are hidden in the dimeric structure. TS also has a monomeric form with distinct function, and the dimeric and the monomeric forms are in equilibrium in humans 37. Therefore, the hidden β‐sheets in the monomeric form may become accessible and may play a role in aggregation processes.
3FJ5: Tyrosine kinase c‐Src (Fig. 1C). It has a role in the mitogen‐activated protein kinase pathway, and in the development of breast cancers in animals and humans 38, 39. It has been shown that the SH3 domain of this protein aggregates to form amyloid fibrils at mild acidic pH values 40. It is suggested that amyloid‐associated microgliosis is strengthened by tyrosine kinase c‐Src activity 41, 42. It has also been noted that mitogen‐activated protein kinase signaling cascade dysfunction in fibroblasts is specific to Alzheimer's disease 43.
2OCT: Stefin B (cystatin B) tetramer (Fig. 1D), an intracellular thiol protease inhibitor. It is known to form amyloid fibrils *in vitro* 44; its role in amyloidogenesis is detailed in 45 and 46.

1HCN: Human chorionic gonadotropin (hCG) (Fig. 1A). It is a placenta‐produced human hormone and applied in numerous pregnancy tests and in legal and illegal drug products, including physical performance‐enhancing and weight‐loss preparations. It is reported to increase β‐amyloid levels in rats 32 and to increase β‐cleavage of an amyloid‐precursor protein 33. Protein hCG also has a role in amyloid β precursor protein expression and modulation in human cells 34, and in protein folding regulation in endoplasmic reticulum 35. We believe that these roles of hCG are closely related to particular geometric properties of its parallel β‐sheets.

## Conclusions

We have demonstrated the validity of three geometric structural selection rules, which identify amyloid fibrils and plaques in the PDB. Additionally, these rules find non‐amyloid soluble proteins, among which we have identified several amyloidogenic ones by scanning the literature. We believe that the great majority of the soluble proteins in the list show also – mostly still undocumented – amyloidogenic properties.

## Author contributions

VG initiated the study and analyzed results. BV created the web interface and the update mechanism. KT designed and programmed the geometric filtering algorithm and fine‐tuned the geometric constraints.

## Conflict of interest

The authors declare no conflict of interest.

## Data availability

The automatically updated web page is available at https://pitgroup.org/amyloid/. The page contains the list of the PDB entries found by our program; each entry is given in graphical form, hyperlinked to the structures at the RCSB PDB site https://www.rcsb.org/pdb.

The Python source code of the software program that generates the PDB_Amyloid list is available at http://uratim.com/amyloid/amyloid_pit.zip.

The page https://pitgroup.org/amyloid/ contains not only the graphical representation of the proteins found but also a list of their PDB codes at https://pitgroup.org/apps/amyloid/amyloid_list.
